# Managing Minor Ailments; The Public’s Preferences for Attributes of Community Pharmacies. A Discrete Choice Experiment

**DOI:** 10.1371/journal.pone.0152257

**Published:** 2016-03-31

**Authors:** Terry Porteous, Mandy Ryan, Christine Bond, Margaret Watson, Verity Watson

**Affiliations:** 1 Health Services Research Unit, University of Aberdeen, Institute of Applied Health Sciences, Polwarth Building, Foresterhill, Aberdeen, AB25 2ZD, United Kingdom; 2 Health Economics Research Unit, University of Aberdeen, Institute of Applied Health Sciences, Polwarth Building, Foresterhill, Aberdeen, AB25 2ZD, United Kingdom; 3 Academic Primary Care, University of Aberdeen, Institute of Applied Health Sciences, Polwarth Building, Foresterhill, Aberdeen, AB25 2ZD, United Kingdom; Chang Gung Memorial Hospital, TAIWAN

## Abstract

**Background:**

Demand for health services continues to rise. Greater use of community pharmacy services instead of medical services for minor ailments could help relieve pressure on healthcare providers in high-cost settings. Community pharmacies are recognised sources of treatment and advice for people wishing to manage these ailments. However, increasing the public’s use of pharmacy services may depend on attributes of pharmacies and their staff. This study aimed to determine the general public’s relative preferences for community pharmacy attributes using a discrete choice experiment (DCE).

**Method:**

A UK-wide DCE survey of the general public was conducted using face-to-face computer-assisted personal interviews. Attributes and levels for the DCE were informed by a literature review and a cohort study of community pharmacy customers. The context for the experiment was a minor ailment scenario describing flu-like symptoms. The DCE choice sets described two hypothetical community pharmacy services; respondents were asked to choose which (if either) of the two pharmacies they would prefer to help them manage symptoms. Data from 1,049 interviews were analysed using an error components logit model. Willingness to pay (WTP), a monetary measure of benefit, was estimated for the different attribute levels.

**Results:**

When seeking help or treatment for flu-like symptoms, respondents most valued a pharmacy service that would improve their understanding and management of symptoms (WTP = £6.28), provided by staff who are trained (WTP (pharmacist) = £2.63: WTP(trained assistant) = £3.22), friendly and approachable (WTP = £3.38). Waiting time, pharmacy location and availability of parking also contributed to respondents’ preferences. WTP for a service comprising the best possible combination of attributes and levels was calculated as £55.43.

**Conclusion:**

Attributes of a community pharmacy and its staff may influence people’s decisions about which pharmacy they would visit to access treatment and advice for minor ailments. In line with the public’s preferences, offering community pharmacy services that help people to better understand and manage symptoms, are provided promptly by trained staff who are friendly and approachable, and in a local setting with easy access to parking, has the potential to increase uptake amongst those seeking help to manage minor ailments. In this way it may be possible to shift demand away from high-cost health services and make more efficient use of scarce public resources.

## Introduction

Community pharmacies are widely recognised as locations from which people seek advice and treatment for the management of minor ailments. Services range from the provision of advice about lifestyle, drug and non-drug treatments, through to advice on symptoms and sales of products, sometimes self-selected by pharmacy customers, for managing minor ailments. Community pharmacy staff have the capacity to support customers wishing to self-care for minor ailments and, if necessary, refer them onwards to appropriate healthcare professionals for further investigations. Internationally, many countries promote this role.[1–3] The aims of such policies include encouraging people to take responsibility for their own health, reducing demand for more expensive healthcare options (such as appointments with primary care medical practitioners or visits to hospital emergency departments) and promoting efficient use of scarce public resources.

Our previous research demonstrated that for minor ailments characterised by flu-like symptoms or acute diarrhoea, people stated a preference for self-care when managing their symptoms.[4,5] Those people wishing to seek advice preferred to get this from community pharmacies or from their general practitioner (GP). In reality, however, a substantial proportion of emergency department (ED) visits and appointments with GPs in the United Kingdom (UK) are for self-limiting conditions that could have been managed without medical intervention, and a similar pattern has been observed in other countries.[6–11] A recent study in the UK estimated that at least 5% of ED visits and 13% of GP appointments concerned such conditions, and that this might cost the National Health Service (NHS) over £1 billion each year.[6] Demand for high-cost health services in the UK continues to rise[12] despite national initiatives that encourage the public to use alternative healthcare services such as telephone help-lines, websites providing health advice, nurse-led minor illness clinics, walk-in clinics and community pharmacy services such as Minor Ailments Schemes (MAS).[13–16] MAS allow patients who are exempt from paying NHS prescription fees to register with a community pharmacy and receive advice and/or treatment for minor ailments, paid for by the NHS.

It is unclear why community pharmacy services are not used more often for the management of minor ailments. One possibility is that service configurations do not meet potential users’ needs or preferences. Previous research investigating the use of community pharmacies [17] and preferences for managing symptoms of minor ailments [4,5] suggests that a number of factors influence community pharmacy use. Some of these are user characteristics (e.g. age, gender, nature of symptoms, previous experience), while others are attributes of pharmacy services. While these studies tell us something about the service attributes that influence the use of pharmacy services by people with minor ailments, none have estimated the relative importance nor strength of preference for the different attributes.[18]

This study used a discrete choice experiment (DCE),[19] a stated preference method, to obtain information from the public about the relative importance of different pharmacy attributes when they consider how to manage a minor illness. Trade-offs between these attributes and the cost of dealing with symptoms of minor illness at a pharmacy, were estimated as respondents’ willingness-to-pay (WTP). These estimates indicate respondents’ strength of preference for each attribute. Policy makers and service providers could use such information to provide services that better satisfy users’ needs and preferences.

DCEs are used in economics to measure preferences for different attributes of goods and services, including healthcare.[19] Data are collected using a structured questionnaire in which respondents are presented with hypothetical “choice sets” that offer alternative goods or services, described in terms of a set of attributes and the levels of those attributes. Selection of appropriate attributes (and associated levels) for the DCE is crucial to the validity of the final results; best practice guidelines recommend that attributes are informed by literature reviews and qualitative evidence.[20,21] Within each choice set, respondents must state which alternative they prefer; it is assumed that the selected alternative maximises their utility (benefit). Regression analysis provides information on relative importance of attributes and how people trade between them when making their choices. If the cost of a service is included as an attribute then WTP for each attribute can be estimated. Calculation of utility scores demonstrates how respondents value specific combinations of attributes and levels; these utility scores can be used to predict the uptake of different service configurations and also total WTP for a service configuration. DCEs have been used previously to value different ways of managing minor ailments including minor ailment nurses in GP practices,[22] nurse prescribing services for minor ailments,[23] and two studies that compared different service providers.[4,5] Elsewhere, DCEs have been used to value extended roles for pharmacists,[24–26] patient-centred pharmacy services,[27] pharmacy services for managing chronic conditions[28] and electronic prescribing.[29]

Studies valuing patient experiences of healthcare have mostly valued the ‘process’ of care in ways that are quantifiable e.g. length of appointment, profession of healthcare provider or location of services. The ‘softer’ attributes of healthcare such as staff attitudes, consultation styles or patient enablement have seldom been considered.[30] Recent research has highlighted the importance of broadening the ways in which patients’ experiences of healthcare are valued and recommends that future studies are designed with this in mind.[31] Whilst identifying attributes for this DCE we remained alert to any such ‘experiential’ attributes that might be suitable for inclusion.

The aims of this study were: to develop and conduct a DCE to establish the public’s preferences for pharmacy service attributes when managing minor ailments; to establish the trade-offs people are prepared to make to ensure access to their preferred pharmacy service, in terms of WTP; and to predict the likelihood of uptake of pharmacy services with specified combinations of attributes.

## Methods

### Discrete Choice Experiment development

A literature review was conducted to identify factors that were said to influence the public’s use of community pharmacies. This included existing quantitative and qualitative research about preferences for pharmacy service attributes, when managing minor ailments. Findings from the review were supplemented with data from a concurrent cohort study exploring the public’s use of community pharmacies to manage minor ailments.[32] In the cohort study, participants visiting a community pharmacy to seek treatment or advice for a minor ailment completed a structured questionnaire indicating why they had chosen to visit a pharmacy on that occasion. From the literature review and the cohort study, more than 30 factors influencing the public’s use of community pharmacy services for the management of minor ailments were identified (details available on request from the authors). Not all of these were suitable as attributes for the DCE; only factors deemed policy-relevant were considered for inclusion as an attribute, i.e. factors that were plausible, actionable and capable of being traded. To further reduce the number of attributes, where possible, potential attributes were collapsed into broader themes encompassing several of the factors identified. For example, factors concerning staff attitude and rapport were collapsed into one that described ‘friendly and approachable’ staff. Final selection of the attributes and their levels was decided after discussions between the authors and a steering group convened specifically for this study. A cost attribute was included to permit the estimation of WTP for different attributes of pharmacy services. The selected attributes and levels are shown in Table 1.

**Table 1:** Attributes and levels.

ATTRIBUTES	LEVELS
Pharmacy location	• At the local shops
	• In a shopping centre
	• In a supermarket
	• Beside a doctor's surgery
Car parking availability	• Definitely (yes)
	• Probably
	• Unlikely
	• No
Who you are served by	• A pharmacist
	• A trained medicine counter assistant
	• An untrained medicine counter assistant
Attitude of staff	• Friendly and approachable
	• Not friendly and approachable
Questions asked by pharmacy staff about symptoms and/or general health	• Yes
	• No
Understanding of symptoms and how to manage them after speaking to pharmacy staff	• You understand your symptoms better and feel like you know the best thing to do to manage them
	• You don't understand your symptoms better and don't feel like you know the best thing to do to manage them
Waiting time until you can deal with symptoms	• 5 hours
	• 12 hours
	• 1 day
	• 2 days
Cost (UK £)	• £2.50
	• £7.50
	• £15.00
	• £25.00

Combining these eight attributes (four with four levels, one with three levels and three with two levels) resulted in 3,072 different possible pharmacy services, which was too many to ask one respondent. These were reduced using *Macro MktEx* in SAS (Version 9.1) [33] to create a d-efficient design with 48 choice sets. To further reduce the burden on respondents and maximise response rates, these were divided into six sub-groups (blocks), each comprising eight choice sets. Each respondent, therefore, was presented with eight choice sets from the full design. Participants were randomly allocated to receive a questionnaire from one of the six blocks; a quota sampling approach ensured that an approximately equal number of responses were completed for each block.

The context for the DCE was a minor ailment symptom scenario (Table 2). Respondents were asked to imagine that they were experiencing flu-like symptoms, that they could not get a GP appointment for 7 days, and that they did not have the medicines they might need at home. They were then asked to choose between two hypothetical community pharmacies that they might go to when managing the symptoms. Alternatively, they could choose a “do nothing” option if neither pharmacy met their preferences. The levels for this “do nothing” option were defined for respondents (Table 3). A full description of the attributes and levels and a worked example of a DCE choice question (Table 3) were provided to assist with the decision-making.

**Table 2:** Symptom scenario.

Please imagine this situation:
• You have a headache and a fever, your bones are aching, you have a sore throat and your nose feels slightly blocked up. You are still able to do all the things you usually do but are more tired than usual. The symptoms started to appear four days ago and were slightly worse when you woke up this morning.
• A doctor’s appointment is not available for 7 days and you don’t have any of the medicines you might need at home.

**Table 3:** Example of a choice question. Please compare the pharmacies and tick which pharmacy, if any, you would visit.

	Pharmacy A	Pharmacy B	Do nothing
Pharmacy location	In a supermarket	Beside Dr surgery	You go nowhere
Find a car park space nearby	Definitely	No	
Waiting time until you can deal with symptoms	5 hours	1 day	No wait
You are served by	A trained medicine counter assistant	Pharmacist	You don’t speak to anyone
Who is	Friendly and approachable	Not friendly and approachable	
Asks questions about your symptoms and general health	Yes	No	
After speaking with pharmacy staff	You **don’t understand** your symptoms any better and you **don’t feel** like you **know the best** thing to do to manage them	You **understand** your symptoms better and you **feel** like you **know the best** thing to do to manage them	No different
Cost	£7.50	£15.00	£0
	**I would visit pharmacy A**	**I would visit pharmacy B**	**I would do nothing**

Please tick one box

### Questionnaire development

A questionnaire was developed for in-person administration using computer assisted personal interviews (CAPI). In addition to the DCE choice questions, data were collected on respondents’ demographic characteristics including age, gender, health status and household income. The format of these questions was, as far as possible, the same as the 2011 UK Census or other national surveys, to allow reliable comparisons with the UK population. For fuller characterisation of respondents, additional questions collected data on current use of community pharmacies. To test the validity of responses to the DCE questions we included a question that asked respondents if they considered all of the pharmacy attributes that were included when they were making their choices in the DCE.

One of the six versions of the questionnaire was pre-piloted in paper format in August 2012 using cognitive interviews [34] with volunteer members of the public (n = 8), identified opportunistically from a local health service user group, and personal contacts of the research team. This resulted in minor clarifications being made to the instructions for completion.

### Participants

The sampling frame was based on UK Census Output Areas (the lowest geographical level at which census estimates are provided). Researchers from *Ipsos Mori* (www.ipsos-mori.com), a professional research company contracted to undertake the survey, used a door-to-door approach to recruit participants for both the pilot and main surveys. The sample was stratified by geographical region and, additionally, subjected to quota sampling for characteristics that included age, gender and working status.

### Survey piloting and administration

The questionnaire was piloted between 21st October and 6th November 2012 using face-to-face CAPI conducted by trained interviewers from *Ipsos Mori*; data were collected from 157 respondents using all six versions (blocks) of the questionnaire. Based on the pilot findings, minor alterations were made to the wording and format of the questionnaire. The main survey was administered by *Ipsos Mori* between 4th and 24th March 2013 using the same CAPI method and with a target sample size of 1000.[35] Data were delivered in an SPSS database (Version 20). The questionnaire is available on request from the authors.

### Data analysis

Analysis of the DCE is based on random utility theory.[36] In this study, an error components logit model was estimated using STATA (Version 12) thus allowing for multiple observations from individuals. In common with most DCEs, the systematic utility *V* of pharmacy alternative *j* is a linear and additive function of the pharmacy attributes and levels with the categorical variables effects coded.

$$Vj=\alpha +\beta_{1}ShopCentre+\beta_{2}Supermarket+\beta_{3}Doctor+\beta_{4}LocalShop+\beta_{5}ParkProbably+\beta_{6}ParkUnlikely+\beta_{7}ParkNo+\beta_{8}ParkDefinitely+\beta_{9}Time+\beta_{10}TMCA+\beta_{11}UTMCA+\beta_{12}Attitude+\beta_{13}Questions+\beta_{14}Understand+\beta_{15}Cost$$ (1)

α represents an alternative specific constant indicating the preference for visiting a pharmacy (as opposed to doing nothing). The sign of the coefficients (β_1_ to β_15_) indicates whether a change in the attribute level has a positive or a negative effect on utility of a pharmacy to respondents.

The unit of measurement must be considered when interpreting the regression results; β_9_ represents the effect on pharmacy choice of a *one hour* increase in waiting time until symptoms can be dealt with, and β_15_ the effect of a *£1* increase in the cost of dealing with symptoms. The coefficients for categorical variables are interpreted as the effect of *the presence* of the attribute level on the utility of the pharmacy, e.g. β_11_ represents the effect on utility of being seen by an untrained medicines counter assistant.

Utility, WTP and probabilities of uptake were calculated for pharmacy services with specified attribute levels. Utility (V) was calculated using Eq 1. WTP for marginal changes in the attributes was estimated using Eq 2, the ratio of the coefficient for the attribute of interest (β_x_) to the negative of the cost coefficient (β_cost_):

$$WTP=\frac{\beta_{x}}{-\beta_{cost}}$$ (2)

Probability of uptake for a hypothetical pharmacy service with utility *V*_*i*_ from a set of actions including alternative available pharmacy services and doing nothing were estimated using the logit Eq 3.

$$P\left(V_{i}\right)=\frac{e^{Vi}}{\sum_{j=1}^{J}\;e^{Vj}}$$ (3)

### Research ethics

The research team had no direct contact with participants in this survey, and their identities were unknown to the team. Ethical approval by an NHS research ethics committee was not required. The College Ethics Review Board at the University of Aberdeen advised that it was the responsibility of *Ipsos Mori*, the company conducting the research, to ensure that the survey was conducted in an ethical manner. *Ipsos Mori* complies with a number of relevant industry quality standards including ISO 27001:2005, the international standard for information security which governs the transfer, storage and destruction of personal data.

## Results

From the 3,885 approaches made to eligible individuals, 1,049 interviews were completed (27.0% acceptance rate). The mean age of respondents was 49 years (SD:18.7), 50.9% were female (n = 534) and the majority (71.9%, n = 754)) self-rated their health as good or very good. All but 99 respondents (9.4%) had visited a pharmacy at least once in the previous six months, mainly for prescription dispensing services. Respondents’ main sources of non-prescription medicines were pharmacies (47.1%, n = 494) and supermarkets (42.1%, n = 442). Table 4 shows further details of respondents’ characteristics compared with other UK statistics.

**Table 4:** Respondent characteristics (N = 1049) compared to UK population.

		*Respondents*		*UK*	
		*%*	*n*	*%*	*p-value*
Gender^1^	Male	49.1	515	49.2	p = 0.805^2^
	Female	50.9	534	50.8	
Age band^1^	18–29	18.7	195	20.3	p = 0.073^3^
	30–44	25.6	266	25.3	(of those
	45–59	23.2	242	25.3	responding)
	60–74	22.0	229	19.0	
	75 and over	10.5	109	10.1	
	Missing	-	(8)	-	
General	Very good	30.5	320	47.2	p < 0.001^3^
health	Good	41.4	434	33.9	
Status^1^	Fair	21.0	220	13.3	
	Bad	5.8	61	4.4	
	Very bad	1.3	14	1.3	
Annual	Up to £5,199 per year	7.6	52	2.0	p <0.001^3^
Income^4^	£5,200 to £10,399 per year	16.3	111	6.9	(of those
	£10,400 to £15,599 per year	15.8	108	12.9	responding)
	£15,600 to £20,799 per year	12.0	82	12.9	
	£20,800 to £25,999 per year	15.1	103	10.9	
	£26,000 to £31,199 per year	7.9	54	8.9	
	£31,200 to £36,399 per year	7.9	54	7.9	
	£36,400 to £51,999 per year	10.4	71	15.8	
	£52,000 and above per year	14.6	100	21.8	
	Prefer not to say	-	(314)	-	
UK Region^1^	North East England	5.0	52	4.1	p = 0.158^3^
	North West England	11.5	121	11.1	
	Yorkshire/Humberside	7.1	75	8.3	
	West Midlands	7.3	77	8.9	
	East Midlands	6.9	72	7.2	
	East of England	10.3	108	9.3	
	South West England	9.8	103	8.4	
	South East England	11.7	123	13.7	
	London	13.1	137	13.1	
	Wales	4.6	48	4.8	
	Scotland	9.3	98	8.3	
	Northern Ireland	3.3	35	2.9	
Usual source	Pharmacy/chemist shop	47.1	494		
of OTC^5^	Supermarket	42.1	442		
medicines^6^	Other	8.4	88		
	Never use OTCs	2.4	25		
Visits to a	0	9.4	99		
pharmacy in	1–5	48.4	508		
past 6-months^5^	6–10	28.7	301		
	11–15	7.7	81		
	More than 15	5.7	60		
Main reason for	Prescription dispensing	75.3	789		
pharmacy visits^5^	Buying OTC medicines	16.2	170		
	Other	5.4	58		
	Never visit pharmacies	3.1	32		

^*1*.^ *Data from 2011 Census*

^2.^ *Binomial test*

^3^ *Chi square test*

4 *Data from Dept*. *for Work & Pensions “Family Resources Survey 2012 to 2013”*

5 *OTC stands for over the counter*

6 *No national data available for comparison*

Results of the regression analysis are presented in Table 5. Theoretical validity of the DCE was demonstrated by the fact that the coefficients for cost and time were significant and negative, i.e. respondents preferred to pay less money and wait less time to deal with symptoms. Sixty-one percent of respondents reported always considering all attributes when making their choices. All attributes had levels that were statistically different from zero indicating that all attributes contributed to respondents’ preferences.

**Table 5:** Results from regression analysis of Discrete Choice Experiment data and marginal willingness to pay.

Variable	Regression coefficient (β)	P value	Willingness to pay (95% CI^a^)
**Constant term**^b^			
Alternative specific constant	2.288	<0.001	£38.31 (33.04, 43.58)
**Pharmacy location**			
Local shops^c^	0.119	0.001	£2.00 (0.81, 3.20)
In a shopping centre	-0.171	<0.001	-£2.86 (-3.84, -1.88)
In a supermarket	-0.080	0.009	-£1.34 (-2.33, -0.35)
Beside a Dr’s surgery	0.131	<0.001	£2.20 (1.18 3.21)
**Car parking availability**			
Definitely^c^	0.147	<0.001	£2.47 (1.24, 3.70)
Probably	0.020	0.527	£0.33 (-0.70, 1.36)
Unlikely	-0.078	0.012	-£1.31 (-2.33, -0.29)
No	-0.089	0.003	-£1.49 (-2.45, -0.53)
**Who you are served by**			
Pharmacist^c^	0.157	<0.001	£2.63 (1.72, 3.53)
A trained medicine counter assistant	0.192	<0.001	£3.22 (2.38, 4.06)
An untrained medicine counter assistant	-0.349	<0.001	-£5.84 (-6.69, -5.00)
**Attitude of staff**			
Not friendly & approachable^c^	-0.202	<0.001	-£3.38 (-3.92, -2.85)
Friendly & approachable	0.202	<0.001	£3.38 (2.85, 3.92)
**Questions asked by staff**			
No^c^	-0.090	<0.001	-£1.52 (-2.02, -1.02)
Yes	0.090	<0.001	£1.52 (1.02, 2.02)
**Understanding of symptoms/management**			
No better understanding^c^	-0.375	<0.001	-£6.28 (-6.90, -5.66)
Better understanding	0.375	<0.001	£6.28 (5.66, 6.90)
**Waiting time** (*time to deal with symptoms in hours)*	-0.014	<0.001	-£0.23 per hour (-£0.27, -£0.19)
**Cost** (*cost of dealing with symptoms in £*)	-0.081	<0.001	
**SD of Alternative specific constant**	3.892	<0.001	
**Number of individuals**	1049		
**Number of observations**	25176		
**Log-likelihood**	-3542.479		
**Akaike Information Criterion**	13114.96		

a 95% confidence intervals are calculated using the delta method

b The coefficient for the constant is used to estimate the preference for doing something to manage symptoms (rather than doing nothing)

c The coefficients for the base cases of the effects coded categorical variables are calculated as the negative of the sum of the coefficients for the other levels. The standard errors are the mean of the other standard errors. The p-values are calculated from the derived coefficients and standard errors

For this flu-like symptom scenario, respondents revealed a preference for visiting a pharmacy to help manage symptoms, rather than doing nothing (as indicated by the positive and significant constant), and were willing to pay around £38 (95%CI: £33.04-£42.58) to do so. The marginal change in an attribute that was most important to respondents (i.e. had the coefficient of greatest magnitude) was whether or not the pharmacy visit would give them a better understanding of their symptoms and how to manage them; WTP for this was valued at £6.28 (95%CI: £5.66-£6.90). Being served by a trained staff member (pharmacist or medicine counter assistant), and friendliness/approachability of pharmacy staff were also valued relatively highly by respondents. To reduce waiting time before they could deal with symptoms by one day, respondents were willing to pay around £5.52 (95%CI: £4.56-£6.48). The most preferred locations for pharmacies were at the local shops or next to the GP surgery; pharmacies in shopping centres and supermarkets reduced WTP. Likelihood of parking also contributed to preferences; locations with a better chance of parking were more highly valued.

Based on these regression results, utility scores, WTP and probability of uptake were calculated for the “best” possible pharmacy (greatest utility), the “worst” pharmacy (least utility) and the “do nothing” option (Table 6). In a world where only these three alternatives exist, the probability that respondents experiencing the symptom scenario (Table 2) would use the “best” pharmacy service was 94.7%, the “worst” pharmacy service, 1.3%, and “doing nothing”, 4%. This suggests that respondents would be more likely to “do nothing” about symptoms than use the “worst” pharmacy service.

**Table 6:** Utility scores, willingness-to-pay and probability of uptake for ‘best pharmacy’, ‘worst pharmacy’ and ‘do nothing’ alternatives.

	Best pharmacy	Worst pharmacy	Do nothing
Pharmacy location	Local shops	Shopping centre	You go nowhere
Find a car park space nearby	Definitely	No	
Waiting time until you can deal with symptoms	5 hours	2 days	No wait
You are served by	Pharmacist	Untrained medicine counter assistant	You don’t speak to a health professional
Who is	Friendly and approachable	Not friendly and approachable	
Asks questions about your symptoms/general health	Yes	No	
After speaking with pharmacy staff	You understand your symptoms better and you feel like you know the best thing to do to manage them	You don’t understand your symptoms any better and you don’t feel like you know the best thing to do to manage them	No different
**Willingness to pay (95% CI)**	**£55.43 (49.24, 61.62)**	**£5.76 (£0.30, £11.24)**	**£0**
Cost	£2.50	£25.00	£0
**Utility score (95% CI)**	**3.16 (2.79, 3.53)**	**-1.15 (-1.50, -0.80)**	**0**
**Probability of uptake**	**94.7%**	**1.3%**	**4%**

## Discussion

These findings indicate that the attributes of a community pharmacy and its staff may influence people’s decisions about which pharmacy they would visit to seek treatment and advice for minor ailments. Specifically, pharmacy customers value being better informed about their symptoms and how to manage them, as well as pharmacy staff that are trained and approachable. Consistent with previous research, attributes relating to access and convenience (in this study: waiting time, location and availability of parking) also influenced preferences. [32]

This is the first published DCE exploring the public’s relative preferences for community pharmacy attributes in the management of minor ailments. The study was conducted in the UK which has publicly funded healthcare (the NHS); these results may, therefore, have limited generalizability to other healthcare systems. This DCE was undertaken in the context of one specific minor ailment. Understanding how people prefer to manage flu-like symptoms is important because self-limiting symptoms of this type are amongst the most common that people present with at their GP.[6] It is possible, however, that our findings may not be generalizable to other minor ailments, although two previous DCEs found that preferences for managing flu-like symptoms and a minor stomach upset were similar.[4,5] The main risk of bias in this type of study is likely to be from sampling bias, particularly since the acceptance rate was relatively low at 27%. We aimed to minimise any such bias by using a stratified quota sampling strategy to ensure that the sample was representative of the population on key indicators (geographical location, age, gender, working status). Our respondents were similar to the UK population in terms of age, gender and location, however, people self-reporting “very good” health and those in higher income brackets were somewhat under-represented. Respondents’ use of community pharmacies was consistent with findings from previous research.[37,38] We used the “gold standard” survey mode for contingent valuation studies (face-to-face interview) [39,40] and the potential for interviewer bias was minimised by the use of CAPI technology and trained interviewers. While we were able to demonstrate theoretical validity of the DCE, we were unable to test external validity; the hypothetical nature of DCEs means that we cannot observe whether or not respondents’ actual behaviour is consistent with their stated preferences. Researchers acknowledge that being able to demonstrate external validity of DCEs is an important issue, but methodological work in this area has been limited [41]; devising methods to test external validity should be prioritised in future work.

Published research has described many of the factors that people say influence their decisions to use a community pharmacy to help them manage minor ailments.[17,42–45] Some of those factors are amenable to change through policy (e.g. pharmacy location); others are less mutable (e.g. customers’ personal characteristics). While it is important to recognise that decision-making in minor illness is multi-factorial, scarce resources may mean that when designing pharmacy services to meet the public’s needs and preferences, practitioners and policy makers wishing to change health-seeking behaviour must concentrate their resources on relatively few of those factors.

Previous studies such as the above mentioned are limited by their largely descriptive nature; none was able to quantify the relative importance of the factors that influenced participants’ decisions to visit pharmacies and how they might trade between them. This DCE has gone beyond such studies by quantifying the relative value that the UK public places on different aspects of pharmacy services for the management of minor ailments, allowing prioritisation of potential service developments. One recent Australian study used a DCE to explore the preferences of people with chronic conditions for pharmacy attributes.[28] The findings were similar to those reported here; respondents with chronic conditions valued informative, person-centred services with easy access.

In our study, provision of information that allowed participants a better understanding of their condition, and trained staff with an approachable attitude were the most highly valued attributes. These findings may seem unsurprising and, some would argue, describe the type of service that pharmacies should be providing as standard. However, research conducted in parallel with this study revealed that the public is slightly less satisfied with consultations and advice-giving in pharmacies when compared with GP practices.[32] Other research, conducted over a relatively prolonged period, also suggests that the content of communication between pharmacy staff and customers could be improved.[46–49] The current study reinforces the need to invest in ensuring that all community pharmacy staff are suitably trained; they should be able to provide the information customers need to understand and manage their symptoms, be able to communicate this in a way that is accessible to customers, and in a friendly and approachable way. Making customers aware of a staff members’ level of training may also add value to the service.

The relatively high value that respondents placed on the experiential attributes of pharmacy services (improved understanding of symptoms and friendly and approachable staff attitudes) demonstrates the importance of valuing the broader patient experience. This is consistent with the findings of another study, which mapped out a wide range of concepts that contributed to patients’ experiences of health services.[31] It concluded that this diversity of patient experience must be attended to when developing health services.

Encouraging better use of community pharmacies by members of the public when managing minor ailments could be achieved through provision of services that more closely meet their needs and preferences. In this way, it should be possible to moderate demand for high-cost services in GP surgeries and EDs. In the UK, for example, the recent Keogh report for NHS England identified community pharmacy as an ‘*underutilised resource*’ that could make a substantial contribution to reducing demand on other health services.[50]

Our findings also provide an insight into customers’ preferred locations of community pharmacies; those that are located at the local shops or the GP practice, and that have a high chance of parking were most highly valued. This is consistent with recent research which demonstrated that convenience and shorter travel distances are amongst the most important influences when people choose where to seek advice for managing their minor ailments.[32]

## Conclusion

Shifting demand for minor ailment consultations away from high-cost settings, such as GP practices and EDs is important, and likely to lead to more efficient resource use in the provision of primary and secondary healthcare. The findings of this study suggest that attributes of community pharmacies and their staff may have an impact on uptake of services for the management of minor ailments. Those that adhere more closely to the type of service most valued by respondents in this DCE are likely to encourage more people to use their pharmacies when managing minor ailments. To achieve this it is important that all pharmacy staff are trained and that the public knows that they are trained. It is also essential that all staff communicate effectively with customers, using their knowledge to help customers understand and manage their symptoms. In addition, future plans for the location of community pharmacies must always incorporate convenient access arrangements. It is only when community pharmacies are designed around patient preferences that there will be a shift away from EDs and general practices for the management of minor ailments.
